# Macroeconomic dataset for generating macroeconomic volatility among selected countries in the Asia Pacific region

**DOI:** 10.1016/j.dib.2017.11.015

**Published:** 2017-11-08

**Authors:** Yee Peng Chow, Junaina Muhammad, Bany Ariffin Amin Noordin, Fan Fah Cheng

**Affiliations:** a Putra Business School, Universiti Putra Malaysia, 43400 Serdang, Malaysia; b Faculty of Economics and Management, Universiti Putra Malaysia, 43400 Serdang, Malaysia

**Keywords:** Macroeconomic, Volatility, Asia Pacific

## Abstract

This data article provides macroeconomic data that can be used to generate macroeconomic volatility. The data cover a sample of seven selected countries in the Asia Pacific region for the period 2004–2014, including both developing and developed countries. This dataset was generated to enhance our understanding of the sources of macroeconomic volatility affecting the countries in this region. Although the Asia Pacific region continues to remain as the most dynamic part of the world's economy, it is not spared from various sources of macroeconomic volatility through the decades. The reported data cover 15 types of macroeconomic data series, representing three broad categories of indicators that can be used to proxy macroeconomic volatility. They are indicators that account for macroeconomic volatility (i.e. volatility as a macroeconomic outcome), domestic sources of macroeconomic volatility and external sources of macroeconomic volatility. In particular, the selected countries are Malaysia, Thailand, Indonesia and Philippines, which are regarded as developing countries, while Singapore, Japan and Australia are developed countries. Despite the differences in level of economic development, these countries were affected by similar sources of macroeconomic volatility such as the Asian Financial Crisis and the Global Financial Crisis. These countries were also affected by other similar external turbulence arising from factors such as the global economic slowdown, geopolitical risks in the Middle East and volatile commodity prices. Nonetheless, there were also sources of macroeconomic volatility which were peculiar to certain countries only. These were generally domestic sources of volatility such as political instability (for Thailand, Indonesia and Philippines), natural disasters and anomalous weather conditions (for Thailand, Indonesia, Philippines, Japan and Australia) and over-dependence on the electronic sector (for Singapore).

**Specifications Table**

**Table:** 

Subject area	Economics
More specific subject area	Macroeconomics
Type of data	Figures, table and Excel files
How data was acquired	Data are acquired from International Financial Statistics (IFS) published by the International Monetary Fund (IMF), World Development Indicators (WDI) by the World Bank, Federal Reserve Economic Data (FRED) by the Federal Reserve Bank of St. Louis, Economic and Social Commission for Asia and the Pacific (ESCAP) Statistical Database by the United Nations, the Organization for Economic Co-operation and Development (OECD), central banks and Department of Statistics (DOS) of each sample country
Data format	Aggregated, processed
Experimental factors	The sample was extracted by merging information from IFS, WDI, FRED, ESCAP, OECD, central banks and DOS. Sample construction involved converting the raw data collected from the various sources into either growth rates or ratios.
Experimental features	The macroeconomic data series represent three broad categories of indicators that can be used to proxy macroeconomic volatility. They are indicators that account for macroeconomic volatility (i.e. volatility as a macroeconomic outcome), domestic sources of macroeconomic volatility and external sources of macroeconomic volatility.
Data source location	Malaysia, Thailand, Indonesia, Philippines, Singapore, Japan and Australia
Data accessibility	Data are available within this article

**Value of the data**

• Macroeconomic volatility may have potential destabilizing effects on a country's or region's economic growth due to its impact on various economic activities such as production, investment and financing. Therefore, this dataset offers an opportunity to conduct volatility studies in the context of developing and developed countries in the Asia Pacific region to assess the effect of macroeconomic volatility on areas such as development economics, corporate finance and banking.
• It is important for any volatility studies to identify the source or type of volatility before assessing the impact of volatility [1]. Therefore, this dataset allows volatility studies to address the multidimensional aspects of macroeconomic volatility.
• This dataset may have important managerial implications since managers of firms often see risk or volatility as multidimensional and adopt an assortment of risk measures in their corporate decisions [2].
• This dataset may also be useful for financial policy makers, monetary authorities and financial institutions because the identification of the sources of macroeconomic volatility facilitates the formulation of appropriate policies and strategies to deal with the effects arising from volatility in the macroeconomic environment.
• Since this dataset spans over the period 2004–2014, it has significant potential for future volatility studies involving before, during and post-Global Financial Crisis periods.

## 1 Data

**Fig. 1:**
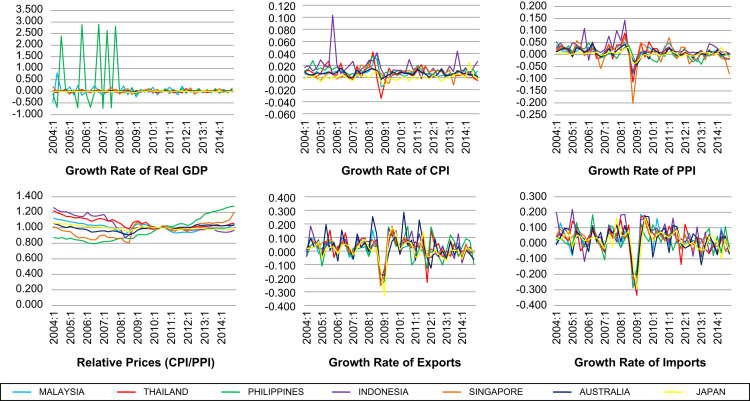
Indicators for volatility as a macroeconomic outcome. Data source: Authors’ own calculations using data from IMF International Financial Statistics.

**Fig. 2:**
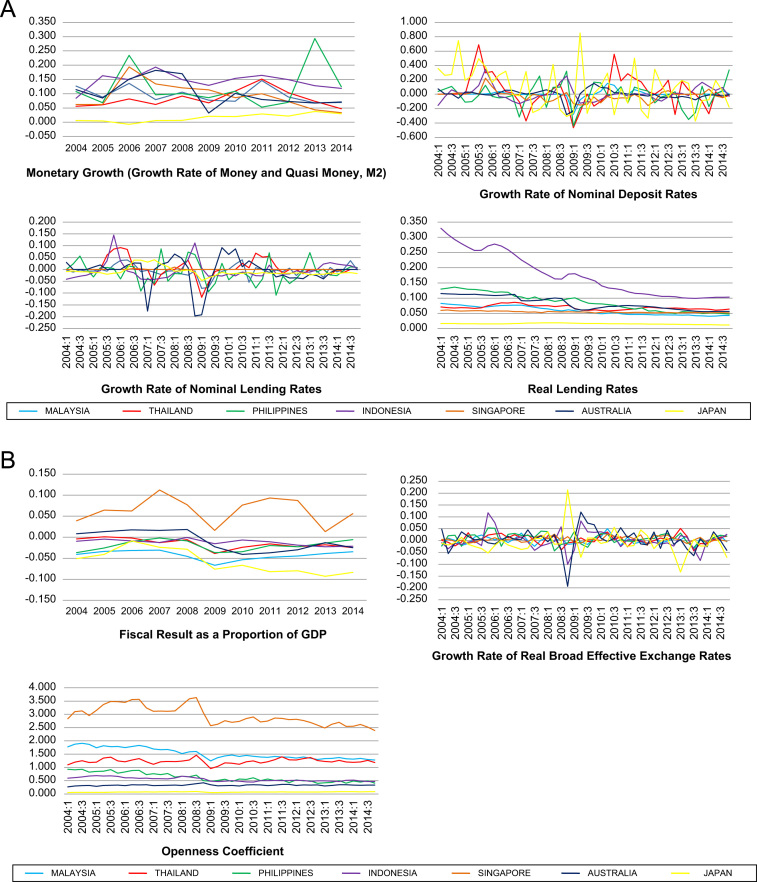
**A.** Indicators for domestic sources of volatility. Data sources: Authors’ own calculations using data from IMF International Financial Statistics and World Bank World Development Indicators. **B.** Indicators for domestic sources of volatility. Data sources: Authors’ own calculations using data from IMF International Financial Statistics, United Nations ESCAP Statistical Database, Federal Reserve Economic Data, OECD and central banks of various countries.

**Fig. 3:**
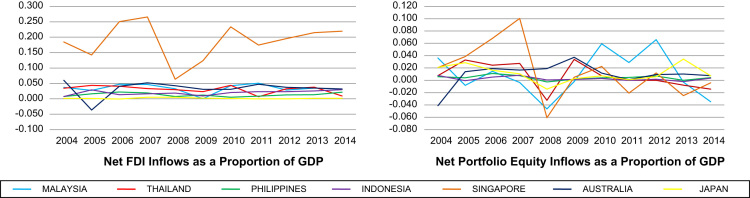
Indicators for external sources of volatility. Data sources: Authors’ own calculations using data from IMF International Financial Statistics, World Bank World Development Indicators and Department of Statistics of various countries.

The data on 15 types of macroeconomic data series are available at the country-level for seven selected countries in the Asia Pacific region for the period 2004–2014. The choice of countries is motivated by several factors. Firstly, they are selected based on data availability. Secondly, these countries have different institutional set-ups such as level of economic development, financial markets and legal origins. In particular, in terms of level of economic development, Malaysia, Thailand, Indonesia and Philippines are regarded as developing countries, while Singapore, Japan and Australia are developed countries. Similarly, while the stock exchanges in Malaysia, Thailand, Indonesia and Philippines are emerging exchanges, those in Singapore, Japan and Australia are more established markets. Moreover, Malaysia, Thailand, Singapore and Australia are countries based on common law, while Indonesia, Philippines and Japan are based on civil law [3], [4]. This diversity gives the opportunity to assess the effects of macroeconomic volatility in different environments. The macroeconomic data series represent three broad categories of indicators that can be used to proxy macroeconomic volatility. They are indicators that account for macroeconomic volatility (i.e. volatility as a macroeconomic outcome), domestic sources of macroeconomic volatility and external sources of macroeconomic volatility. Fig. 1, Fig. 2, Fig. 3 depict the graphs for the macroeconomic data series. Meanwhile, the data for the macroeconomic data series are available in 15 separate Excel spreadsheets, one for each macroeconomic data series. From these graphs, it can be seen that these countries are subject to wide fluctuations arising from various sources of macroeconomic volatility.

## 2 Experimental design, materials and methods

The data cover a sample of seven selected countries in the Asia Pacific region for the period 2004–2014. These macroeconomic data are gathered from multiple reliable sources such as IFS, WDI, FRED, ESCAP, OECD, central banks and DOS, and are converted into either growth rates or ratios.

Firstly, there are six indicators that account for macroeconomic volatility (i.e. volatility as a macroeconomic outcome), namely growth rate of real Gross Domestic Product (GDP), growth rate of Consumer Price Index (CPI), growth rate of Producer Price Index (PPI), relative prices, growth rate of exports and growth rate of imports. GDP growth measures the overall economic condition in a particular country. CPI serves as a proxy for the price of non-tradable goods, while PPI is a proxy for the price of tradable goods. Relative prices is the ratio of CPI to PPI. Exports and imports measure merchandise trades.

Secondly, there are seven indicators for domestic sources of macroeconomic volatility, namely monetary growth, growth rate of nominal deposit rates, growth rate of nominal lending rates, real interest rate, fiscal result as a proportion of GDP, growth rate of real broad effective exchange rates and openness coefficient. Monetary growth and interest rates are indicators for monetary policy. Fiscal result as a proportion of GDP serves as an indicator for fiscal policy. Real broad effective exchange rate growth is an indicator for exchange rate policy, and openness coefficient is an indicator for trade policy.

Thirdly, there are two indicators for external sources of macroeconomic volatility, namely net foreign direct investment (FDI) inflows as a proportion of GDP and net portfolio equity inflows as a proportion of GDP. Both indicators measure capital mobility. Table 1 provides the variable definitions for the macroeconomic series.

**Table 1:** Variable definitions.

Variable definition	Data frequency	Source of Data
*Indicators for volatility as a macroeconomic outcome*		
Growth rate of real GDP	Quarterly	IFS
Growth rate of CPI	Quarterly	IFS
Growth rate of PPI	Quarterly	IFS
Relative prices (measured as CPI over PPI)	Quarterly	IFS
Growth rate of exports FOB^a^	Quarterly	IFS
Growth rate of imports CIF^b^	Quarterly	IFS
*Indicators for domestic sources of volatility*		
Monetary growth (growth rate of money and quasi money, M2)	Annually	WDI
Growth rate of nominal deposit rates	Quarterly	IFS
Growth rate of nominal lending rates	Quarterly	IFS
Real interest rate (measured as nominal lending rate adjusted for inflation as measured by the GDP deflator)	Quarterly	IFS
Fiscal result as a proportion of GDP	Annually	ESCAP, OECD, central banks
Growth rate of real broad effective exchange rates	Monthly	FRED
Openness coefficient (measured as the sum of exports and imports over GDP)	Quarterly	IFS, FRED
*Indicators for external sources of volatility*		
Net FDI inflows as a proportion of GDP	Annually	WDI
Net portfolio equity inflows as a proportion of GDP	Annually	WDI, IFS, DOS

a FOB denotes Free On Board.

b CIF denotes Cost, Insurance and Freight.
